# Relationship between Dietary Habits, Food Attitudes and Food Security Status among Adults Living within the United States Three Months Post-Mandated Quarantine: A Cross-Sectional Study

**DOI:** 10.3390/nu12113468

**Published:** 2020-11-12

**Authors:** Aljazi Bin Zarah, Juliana Enriquez-Marulanda, Jeanette Mary Andrade

**Affiliations:** Food Science and Human Nutrition Department, University of Florida, Gainesville, FL 32611, USA; aljazibinzarah@ufl.edu (A.B.Z.); Juliana.enriquez@ufl.edu (J.E.-M.)

**Keywords:** COVID-19, adults, dietary habits, food attitudes, food security

## Abstract

COVID-19 has disrupted the lives of many and may have influenced dietary habits through factors such as food security status and attitudes. The purpose of this study was to identify dietary habits and their associations with food insecurity and attitudes among adults living in the United States within three months post-mandated quarantine. An online cross-sectional study was conducted from April to June 2020. Participants (*n* = 3133) responded to a 71-item questionnaire regarding demographics (*n* = 7), health information (*n* = 5), lifestyle habits (*n* = 8), dietary habits (*n* = 37), food attitudes (*n* = 8), and food security status (*n* = 6). Frequency counts and percentages were tabulated, and multivariate linear regression was conducted to examine associations using STATA v14 at a statistical significance level of *p* < 0.05. Results showed that most participants indicated no change in dietary habits (43.6–87.4%), yet participants reported increased consumption of sweets (43.8%) and salty snacks (37.4%). A significant positive association for food attitude scores (1.59, 95% CI 1.48 to 1.70; *p* < 0.001) and food security scores (1.19, 95% CI 0.93 to 1.45; *p* < 0.001) on total dietary habit scores was found. Future extensive population studies are recommended to help public health authorities frame actions to alleviate the impact that mandated quarantine has on dietary habits.

## 1. Introduction

Coronavirus, also known as SARS-CoV-2 or COVID-19, is a severe acute respiratory syndrome in which more than 40 million global cases and more than 1.1 million deaths have been identified [1]. In the United States (U.S.), there have been more than 8.2 million cases and 221 thousand reported deaths [2]. During March, the U.S. federal government mandated that all residents and citizens remain in quarantine. Only essential workers such as medical professionals and grocery store personnel were allowed to be physically present at the workplace [3]. In April, the federal government eased restrictions; however, several states, businesses, schools, and other organizations continued to encourage adults to remain in their homes and limit the time spent at establishments to reduce the spread of the virus. As a result of limited economic activity, many businesses closed their doors, which resulted in 14.7% of adults being unemployed in April [4,5]. Projections are that food insecurity will increase by as much as 5.2% due to the rates of unemployment [5,6,7].

Even though these preventative measures were necessary to reduce the virus’s spread, it may have unwillingly shifted dietary habits based on food security status. As models have shown, during a pandemic, individuals reduce their consumption of animal protein, fruits, and vegetables due to the increased cost and availability of these foods [8,9]. Additionally, during high-stress times, attitudes such as boredom or anxiety may also influence dietary habits as foods typically consumed are considered snacks or comfort foods that are typically high in sodium, added sugars, and total fats [10]. This is further observed by the recent COVID-19 studies that have focused on dietary habits [11,12,13,14,15,16] or the influence that attitudes [17,18,19,20] had on dietary habits. Notably, an Italian study showed that participants decreased their consumption of fruits and vegetables (8.7%), with 33.5% of participants stating that this was due to the lower availability of these foods. Additionally, around 46% of participants reported an increase in snacking, 42.5% reported an increase in their consumption of sweetened snacks such as chocolate, ice cream, and desserts, while 23.5% reported an increase in their intake of salty snacks. Furthermore, these dietary habits occurred due to feelings of boredom, fear, anxiety, and stress [16]. 

The effects of COVID-19 on adults’ dietary habits are likely due to food security status and attitudes. However, there is limited evidence that has associated these factors with dietary habits. Therefore, this study identified dietary habits and their associations with food security status and attitudes among adults living in the U.S. within three months post-mandated quarantine. 

## 2. Materials and Methods 

### 2.1. Study Design and Participants 

**Figure 1:**
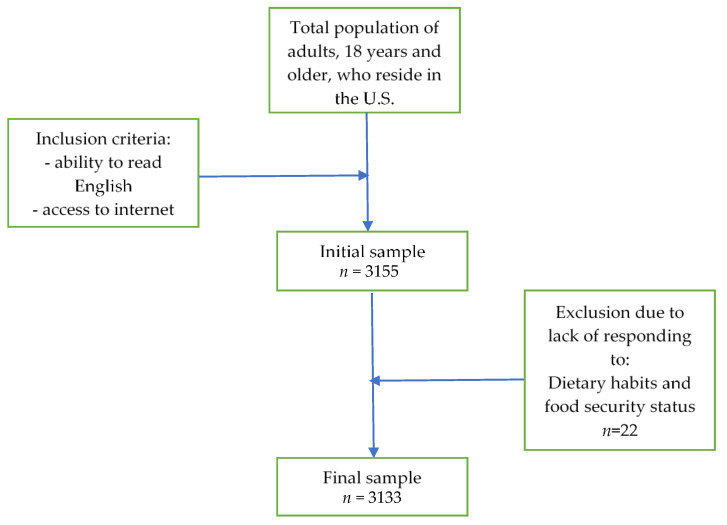
Sample collection chart.

This cross-sectional study was conducted online through Qualtrics^XM^ (Qualtrics, Provo, UT, USA), an online survey platform, from April to June 2020. Recruitment was voluntary and anonymous and occurred through social media platforms and ResearchMatch (NIH CTSA, Nashville, TN, USA). ResearchMatch is a national health volunteer registry created by several academic institutions and supported by the National Institutes of Health as part of the Clinical Translational Science Award (CTSA) program [21]. Adults were eligible to participate if they were above the age of 18 and were able to read in the English language and lived in the U.S. during COVID-19. All subjects gave their informed consent for inclusion before they participated in the study. A total of 3155 adults initially participated; after excluding non-responses regarding dietary habits and food security, the final dataset included 3133 participants (see Figure 1). All study protocols were granted ethical approval by the University of Florida Institutional Review Board # 202001147.

### 2.2. Questionnaire

Participants responded to a 71-item questionnaire regarding demographics (*n* = 7), health information (*n* = 5), lifestyle habits (*n* = 8), dietary habits (*n* = 37), food attitudes (*n* = 8), and food security status (*n* = 6). The researchers (ABZ and JMA) developed this questionnaire with the use of adapted validated instruments. The instrument was assessed for face validity by five adults and modifications were made to enhance clarity (Supplementary File). The total length of time to complete the questionnaire was estimated at 10 minutes. The demographic questions (*n* = 7) included age, sex, race/ethnicity, education level, employment status, geographic location of residence, and time spent at home since COVID-19. Health information questions (*n* = 5) that were self-reported by the participants included current height reported in feet and inches and weight reported in pounds for the researchers to calculate body mass index (BMI) (body mass (kg)/height (m^2^)) and interpreted according to the criteria of the Centers for Disease Control and Prevention [22]. Seven categories were identified: underweight (BMI < 18.5 kg/m^2^), normal weight (18.5 kg/m^2^–< 25.0 kg/m^2^), overweight (25.0 kg/m^2^–BMI < 30.0 kg/m^2^), obesity class 1 (BMI 30.0 kg/m^2^–< 35.0 kg/m^2^), obesity class 2 (BMI 35.0 kg/m^2^–< 40 kg/m^2^), and obesity class 3 (BMI > 40 kg/m^2^) [22]. Additional health questions were about weight changes, health conditions, supplement use, and if participants followed a diet since COVID-19. 

#### 2.2.1. Lifestyle Habits

Lifestyle habit statements (*n* = 8) focused on participants’ physical and social activities during COVID-19. For physical and social activities, participants indicated whether the behaviors increased, decreased, or did not change during COVID-19. To determine total scores, no change resulted in a score of 0. An increase in eating, watching TV, or smoking or a decrease in exercising, physical activity, sleep amount/quality, reading/studying, or socialization resulted in a score of 1 as these were considered unfavorable to health [13,19,23]. If participants indicated a decrease in these activities (e.g., eating) or an increase in these activities (e.g., exercising), it resulted in a score of 2 as these activities were considered favorable to health [13,19,23]. Total scores ranged from 0 to 16.

#### 2.2.2. Dietary Habits

Participants completed a section about dietary habits based on foods/beverages consumed. The foods and beverages listed were based on Dana-Farber’s Cancer Institute Eating Habits Questionnaire [24]. The Cancer Institute questionnaire originally included five food categories—dairy, fruits, vegetables, meats, sweets, baked goods, and beverages—with a total of 61 food/beverage items based on the frequency of consuming those items over the past year. The instrument was modified to reflect six food/beverage categories with 37 items, to reduce potential survey exhaustion from participants [25,26]. For example, instead of listing specific fruits (e.g., oranges, bananas), information was grouped into fresh/frozen or canned fruit. For dietary habits, total scores were determined based on whether the participant selected increased, decreased, or no change in these habits since COVID-19. Selecting no change resulted in a score of 0. Selecting increased from the following food/beverage items resulted in a score of 2 for each item or by selecting decreased results in a score of 1 for each item as these were considered nutrient-dense foods (i.e., low in sodium, added sugars and total fat): milk and yogurt, fresh/frozen/canned fruits and vegetables, chicken and fish, whole grains (e.g., whole wheat/brown bread/rice), water, non-carbonated no added sugar beverages, immune-enhancing beverages, coffee/tea, and protein shakes [27,28]. Selecting increased in the following food/beverage items resulted in a score of 1 for each item or selecting decreased resulted in a score of 2 for each item as these foods were considered energy-dense foods (i.e., high in sodium, added sugars and total fat): cheese, butter/margarine, fruit juice, vegetable/tomato juice, processed meats, red meats, refined grains (e.g., white bread/rice), chips, sweets, alcohol (e.g., beer, wine, spirits), and carbonated added sugar beverages [27,28]. The total scores ranged from 0 to 74 points.

#### 2.2.3. Food Attitudes 

The second section of the survey asked participants to indicate an increase, decrease, or no change (never had these thoughts) in eight statements regarding food attitudes since COVID-19. These statements included eating much more than planned, over-eating, lethargy after eating, and stress behaviors derived from the Yale Food Addiction Scale [29]. The original addiction scale has 16 statements based on a response from 1 (never) to 5 (4 or more times daily) over the past 12 months. This instrument has internal reliability for a single-factor of ∝ = 0.75 based on Kuder–Richardson. It has been validated through convergent, divergent, and incremental methods based on a sample of 1440 participants [29]. Total scores were determined through the identification of increased/decreased or no change reports. If participants had no change in their food attitudes, a score of 0 was provided. An increase in these thoughts was given a score of 2. For a decrease in these thoughts, a score of 1 was provided. Thus, the total scores ranged from 0 to 16. 

#### 2.2.4. Food Security 

Household food security was measured using the validated USDA Food Security Module. The short module includes six questions, ordered by the severity of food insecurity, that ask about a household’s experiences with food insufficiency during the previous twelve months. The survey in this study was adapted to the COVID-19 circumstances. This short-item survey has been validated to represent 97.7% of households and is intended to be answered by a representative of the household [30,31]. This 6-item survey is scored on a scale from 0 to 6, with 0–1 representing high or marginal food security, 2–4 representing low food security, and a score of 5–6 representing even lower food security [30]. The scoring system has been validated by previous studies [30,31]. 

### 2.3. Statistical Analysis 

Frequency counts and percentages were tabulated for demographic variables and dietary habits, food attitudes, and food security scores similarly to a previous study [11]. Multivariate linear regression was conducted to examine the impact of associations of food insecurity and attitudes on dietary habits. This analysis examined the confounding factors (food attitudes and food security status) and isolated the relationship of interest (dietary habits). An additional regression was conducted that focused on the impact of the confounding variables, demographics and lifestyle, on dietary habits [32]. The average dietary habits score was regressed onto demographics, lifestyle habits, food security status, and food attitudes as shown in Table 1. The effect size classification suggested by Cohen [33] was used to present the strength of R^2^, which was classified as small, medium, and large when R^2^ = 0.01, 0.09, and 0.25, respectively [34,35]. Statistical significance was determined at *p* < 0.05. All statistical analyses were conducted using STATA (version 14.0, StataCorp, College Station, TX, USA).

## 3. Results

### 3.1. Study Population 

The sample consisted of 3133 respondents, although not all participants responded to demographic or health statements. For those who responded to these statements, the majority were white (84.5%), female (79.4%), held a bachelor’s or master’s degree (34.2% and 30.3%, respectively), and were employed full-time (43.0%). The sample’s age range varied, with a slight majority between the ages of 30 to 49 years old (30.5%). Half of the participants were married (50.5%), lived in the South Atlantic region (22.9%), lived with at least one person (42.2%), and had stayed in their homes 75% to 95% of the time during the three months within post-quarantine measures (79.4%) (see Table 2). 

#### Health Characteristics and Anthropometrics

For participants who responded to these questions, health characteristics and anthropometrics revealed that based on the calculated body mass index (BMI) kg/m^2^, participants were considered obese (47.0%) or overweight (34.0%). Most participants were not currently on a diet (82.8%), had no weight changes during the pandemic (43.0%), and did not take any supplements (79.3%). Of those using supplements, the majority were taking four or more (36.8%). Participants did indicate that they had at least two medical conditions (30.8%) (see Table 3).

### 3.2. Dietary Habits

**Figure 2:**
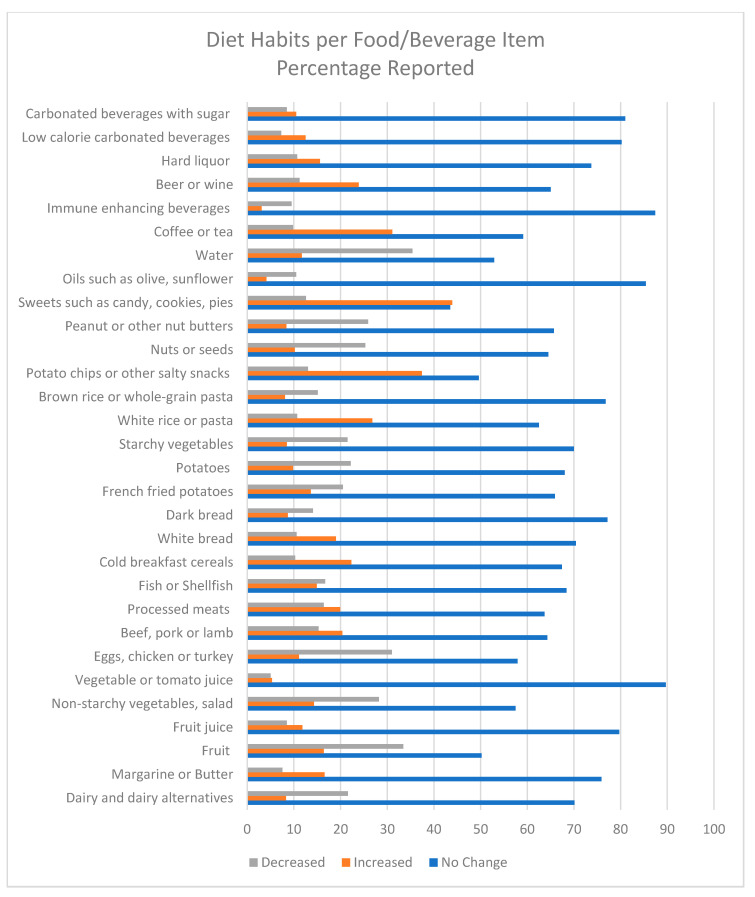
Dietary habits reported by foods/beverages consumed. Data represented as percentages of no-change, increased, or decreased.

On average, the total dietary habits score was 14.39 ± 9.78, with a range of scores from 0 to 53. Most participants indicated no change in dietary habits (43.6–87.4%) for the listed food and beverage items. Some participants, though, reported increased consumption of sweets, including cakes, cookies, and pies (43.8%); potato chips or salty snacks (37.4%); water (35.4%); coffee or tea (31.1%); white rice or pasta (26.8%); alcoholic beverages (23.9% and 15.6%); cold breakfast cereals (22.3%); baked, mashed, or boiled potatoes (22.2%); starchy vegetables (21.6%); beef, pork, or lamb (20.4%); processed meats (20.0%); white bread (19.0%); margarine or butter (16.5%); fruit juice (11.7%); vegetable juice (5.3%); and carbonated beverages with sugar (10.6%). Furthermore, some participants reported decreased consumption of fruit (33.4%); eggs, chicken, or turkey (31%); non-starchy vegetables (28.2%); dairy (21.6%); and fish and shellfish (16.6%). Participants also indicated decreased consumption of nutritious foods such as nut butter (26.0%); nuts or seeds (25.3%); brown rice or whole-grain pasta (15.1%); whole-grain bread (14.1%); and oils (10.7%) (see Figure 2).

### 3.3. Association between Food Security Status and Food Attitudes on Dietary Habits 

Average scores for food attitudes were 2.60 ± 2.99 (minimum score of 0 and maximum score of 12) and for food security were 0.69 ± 1.77 (minimum score of 0 and maximum score of 10). A multivariate linear regression showed a significant positive correlation for food attitudes score (1.59, 95% CI 1.48 to 1.70; *p* < 0.001) and food security score (1.19, 95% CI 0.93 to 1.45; *p* < 0.001) with total dietary habits score, controlling for demographic cofounding factors and the interaction term (Table 4). A significant negative correlation was found for the female sex (−0.97, 95% CI −1.69 to −0.24; *p* = 0.009), race (−0.74, 95% CI −1.1 to −0.37; *p* < 0.001), and age range (−1.01, 95% CI −1.26 to –0.77; *p* < 0.001) with total dietary habits score. A significant positive correlation was found for percentage of time spent at home (−1.25, 95% CI −0.70 to −1.81; *p* < 0.001) with total dietary habits score. This model had a large strength, with an R^2^ of 0.29.

A second multivariate linear regression revealed a significant positive correlation for BMI (0.065, 95% CI 0.02 to 0.11; *p* = 0.006), weight change (0.60, 95% CI 0.11 to 1.09; *p* = 0.017), use of supplements (2.16, 95% CI 1.32 to 3.00; *p* < 0.001), and total activity score (1.14, 95% CI 1.00 to 1.28; *p* < 0.001) with dietary habits score. This model had a large strength, with an R^2^ of 0.37 (see Table 4).

## 4. Discussion

This cross-sectional study demonstrated that dietary habits and their associations between food security status and food attitudes among adults living within the U.S. three months post-mandated quarantine were impacted. For dietary habits, participants reported increased consumption of sweets, red and processed meats, and refined grains and decreased consumption of whole fruits, vegetables, and lean proteins. Additionally, participants reported decreased consumption of whole grain bread, nuts/seeds, and oils. Factors such as female sex, race, and age range had a negative correlation with dietary habits, whereas time spent at home, BMI, weight change, use of supplements, and total activity score positively correlated with dietary habits. 

Despite COVID-19 being responsible for 10.6 million job losses and a rise in the consumer price index of food [4], in this study, participants reported high food security during these times, which is contradictory to other reports [5,36,37]. Results from this study showed that low food security scores were associated with lower dietary habits scores. Therefore, individuals that were considered food secure were less likely to change their dietary habits. These results concur with the United States Department of Agriculture’s (USDA) definition of food security, as food insecurity is characterized by disrupted standard eating patterns and multiple changes in the diet due to minimal resources to access food [38]. High food availability could have also contributed to the high food security scores observed in this study. Even though grocery stores reported less food availability and higher costs of food at the start of the pandemic due to unpreparedness, demand for food slowly stabilized, and prices for food items returned to typical figures [39,40]. For instance, the price for a dozen eggs in New York rose to USD 3.07 by the end of March but gradually decreased to USD 1.97 by mid-April [39]. 

Food attitudes and dietary habits score were positively correlated such that, on average, participants had lower food attitudes and lower dietary habits scores. Lower food attitude scores conveyed a lack of distress towards dietary habits. These results may conflict with those reported by Czeisler et al., who indicated elevated levels of impaired mental health during COVID-19, including a three-fold increase in anxiety disorders and a four-fold increase in depression [41]. However, the results of this study align with those found by Termorshuizen et al., which reported that 49% of the U.S. participants indicated an increased connection with family and friends, which led to adaptive coping skills and positive changes in their mental health during COVID-19 [42]. A study on the changes in dietary habits amid COVID-19 in Spain reported that participants who lived with their family during Spain’s confinement displayed higher adherence to the Mediterranean diet [20]. Since a little over 40% of participants in this U.S. study reported living with at least one person during confinement, it is possible that positive relationships led to better coping skills, which in turn led to fewer impaired food attitudes. 

It was expected that during quarantine, animal protein, fruits, and vegetable consumption would decrease due to lower availability and financial access [9]. However, this study found that participants had reported no change in the intake of these commodities. There was no major variation in dietary patterns aside from increases in the consumption of sweets and salty snacks. Participants were found to have low dietary habits scores, which did not necessarily reflect poor nutritional behaviors but rather no change in the intake of most food items amid COVID-19. These findings are similar to those reported in an Italian sample by Scarmozzino and Visioli, in which most responders (49.6%) did not modify their diets during quarantine [16]. These results might seem contradictory to those found by the International Food Information Council (IFIC), which reported that 8 in 10 Americans changed their dietary habits amid COVID-19 [43]. However, the change in dietary habits in the IFIC’s study was mainly due to increased cooking at home [43]. Furthermore, the IFIC study did not assess the individual’s change in consuming different food items. Regardless, the IFIC’s study and multiple other studies reported an increase in snacking behavior and consumption of comfort foods (e.g., foods high in sodium, added sugars, and/or total fats) [11,13,14,16,18,19,20,43,44], which is consistent with the increased consumption of sweet and salty snacks observed in this study. 

Participants reported a decrease in the consumption of fruits, non-starchy vegetables, dairy, fish, shellfish, eggs, and white meat (chicken or turkey). Additionally, participants consumed more red meat (beef, lamb, pork), and caffeinated and alcoholic beverages. Laguna et al. found that Spanish consumers also decreased their purchases of fish and shellfish as these had a reduced shelf-life and had a higher price [45]. However, in comparison to the results of this study, Laguna et al. revealed that Spanish consumers increased their intake of fruits, vegetables, eggs, and dairy and decreased their consumption of alcoholic beverages and sweets [45]. These effects could be explained by the differences in dieting in Spain compared to the U.S. While multiple Spanish studies found an increase in the adherence to a Mediterranean diet [14,20], participants in this study reported not following any diet (82.8%). 

In this study, participants (38.0%) reported an increase in their weight. This effect could be attributed to the increased consumption of sweets (43.8%) and salty snacks (37.4%). Furthermore, most participants were identified to be overweight (34.0%) or obese (47.0%), with two or more medical conditions (30.8%). BMI had a significant positive correlation with dietary habits, whereas medical conditions had no effect. These results agree with the findings of another study, which revealed that a higher BMI was associated with increased weight gain, lower consumption of fruits and vegetables, and an elevated intake of meat and alcoholic beverages during quarantine [13]. Another factor found to influence dietary habits was total activity. This study found that while exercise decreased (34.5%), the use of electronic devices increased (71.9%). Higher technology use was expected as social distancing orders transitioned most social and work life to a virtual format. Multiple studies have outlined the relationship between sedentary behaviors and weight gain during COVID-19 [20,23,46]. The decrease in exercise could further contribute to the weight gain which some participants experienced during confinement.

### Strengths and Limitations

To our knowledge, this is the first study to focus on identifying the dietary habits of adults living in the U.S. during COVID-19 post-mandated quarantine. Even though over 3000 participants from across the U.S. participated in this study, the investigators were unable to generalize the results due to the limited demographic variability. As this study was conducted online, only individuals with access to WiFi and a technological device were able to participate. This inadvertently led to selection bias [25]. Self-reporting bias may have been present in the participants’ responses as participants were not forced to respond to each question [25,47]. This bias may have been present in one of two ways: recall bias or social desirability bias. When answering questions regarding dietary habits, attitude or lifestyle behavior recall bias, or error in recalling a past event, may have inhibited accurate responses [25,47]. Additionally, questions concerning more sensitive topics such as food security status, weight, and health conditions may have resulted in social desirability bias or inaccurate reporting due to desired approval [25,47]. 

## 5. Conclusions

The present study was designed to identify dietary habits and their associations with food insecurity and attitudes among adults living in the United States within three months post-mandated quarantine. The research has shown a significant correlation between food security status and food attitudes. Home confinement directly affects lifestyle patterns, including dietary habits, access to food, and food attitudes. This interruption of a routine lifestyle led to non-nutritious food consumption such as those high in sodium, added sugars, and total fats. 

COVID-19 continues to evolve globally, possibly having a prolonged effect on the relationship between dietary habits, food security status, and food attitudes, as shown in the current study. Maintaining consistent dietary habits is difficult during confinement, as the availability of food varies. Future extensive population studies are recommended in the U.S. to help public health authorities to frame actions to alleviate the impact that mandated quarantine has on dietary habits. 

## Figures and Tables

**Table 1:** Model for regression analysis.

Y_1_ = b_0_ + b_1_X_1_ + b_2_X_2_ +… + b_k_X_k_	
where	
Y_1_ represents	Dietary habits
b_0_, b_1_ and b_k_ represent	Estimate regression parameters
X_1_ X_2_ and X_k_ represent	k predictors (demographics, lifestyle habits, food attitudes, and food security status)

**Table 2:** Participants’ demographics.

Variables	No. of Responses (%)
Sex	*N* = 3101
Male	614 (19.8%)
Female	2462 (79.4%)
Other	25 (0.8%)
Race/Ethnicity	*N* = 3099
African American	158 (5.1%)
Asian	89 (2.9%)
White	2620 (84.5%)
Hispanic	87 (2.8%)
Native American	11 (0.4%)
Other	134 (4.3%)
Age	*N* = 3106
18–24 years	206 (6.6%)
25–29 years	300 (9.7%)
30–49 years	946 (30.5%)
50–59 years	548 (17.6%)
60–69 years	647 (20.8%)
>70 years	459 (14.8%)
Education level	*N* = 3106
No schooling completed	2 (0.1%)
Some high school, no diploma	9 (0.3%)
High school graduate, diploma, or the equivalent (for example, GED)	71 (2.3%)
Some college credit, no degree	351 (11.3%)
Trade/technical/vocational training	63 (2.0%)
Associate degree	189 (6.1%)
Bachelor’s degree	1062 (34.2%)
Master’s degree	942 (30.3%)
Professional degree	137 (4.4%)
Doctorate degree	280 (9.0%)
Current employment status	*N* = 3103
Full time	1333 (43.0%)
Part time	361 (11.6%)
Unemployed	542 (17.5%)
Other	867 (27.9%)
Marital status	*N* = 3103
Married	1567 (50.5%)
Single	908 (29.3%)
Widowed	125 (4.0%)
Divorced	401 (12.9%)
Other	102 (3.3%)
People live in the household besides yourself	*N* = 3114
None	630 (20.2%)
1	1314 (42.2%)
2	525 (16.9%)
3	363 (11.7%)
4	146 (4.7%)
5 or more	104 (3.3%)
Did not respond	32 (1.0%)
Currently staying at home ×% of the time	*N* = 3105
Less than 25%	0 (0%)
25–49%	132 (4.3%)
50–75%	404 (13.0%)
75–95%	2465 (79.4%)
Never left the house	104 (3.3%)
Residence	*N* = 3098
New England (Connecticut, Maine, Massachusetts, Rhode Island, Vermont)	119 (3.8%)
Mid-Atlantic (New Jersey, New York, Pennsylvania)	393 (12.7%)
South Atlantic (Delaware, Florida, Georgia, Maryland, North Carolina, South Carolina, Virginia, Washington DC, West Virginia)	710 (22.9%)
East North Central (Illinois, Indiana, Michigan, Ohio, Wisconsin)	573 (18.5%)
East South Central (Alabama, Kentucky, Mississippi, Tennessee)	268 (8.7%)
West North Central (Iowa, Kansas, Minnesota, Missouri, Nebraska, North Dakota, South Dakota)	242 (7.8%)
West South Central (Arkansas, Louisiana, Texas)	161 (5.2%)
Mountain (Arizona, Colorado, Idaho, Montana, Nevada, New Mexico, Utah, Wyoming)	202 (6.5%)
Pacific (Alaska, California, Hawaii, Oregon, Washington)	430 (13.9%)

Note. GED = General Educational Development.

**Table 3:** Participants’ general health characteristics and anthropometrics.

Variables	No. of Responses (%)
BMI (kg/m^2^)	*N* = 3040
< 18	37 (1.2%)
18.5–24.9	538 (17.7%)
25–29.9	1033 (34.0%)
30–34.9	733 (24.1%)
35–39.9	357 (11.7%)
40–44.9	194 (6.4%)
>45	146 (4.8%)
Weight change	*N* = 3110
No change	1336 (43.0%)
Increased	1182 (38.0%)
Decreased	592 (19.0%)
Activity	*N* = 3103
No change	1102 (35.5%)
Increased	1326 (42.7%)
Decreased	675 (21.8%)
Tried a diet	*N* = 3123
No	2587 (82.8%)
Yes	536 (17.2%)
Nutritional supplement intake	*N* = 3119
No	2474 (79.3%)
Yes	645 (20.7%)
Supplements currently taking	*N* = 646
Multi-vitamin	47 (7.3%)
Vitamin B complex	5 (0.8%)
Vitamin C	22 (3.4%)
Vitamin D	26 (4.0%)
Other	47 (7.3%)
Two supplements	150 (23.2%)
Three supplements	111 (17.2%)
Four or more supplements	238 (36.8%)
Medical conditions	*N* = 1960
Cancer	24 (1.2%)
Depression	274 (13.9%)
Diabetes (high blood sugar)	52 (2.7%)
Diverticulosis/Diverticulitis	10 (0.5%)
Gastric reflux	80 (4.1%)
Heart disease	143 (7.3%)
IBS/D	47 (2.4%)
Liver disease (cirrhosis, fatty liver)	4 (0.2%)
Lung disease	17 (0.9%)
Nausea/Vomiting	9 (0.5%)
Other	294 (15.0%)
2 conditions	604 (30.8%)
3 or more conditions	402 (20.5%)

Note. BMI = Body Mass Index; IBS/D = Irritable Bowel Syndrome/Disease.

**Table 4:** Multivariable associations and total dietary habits score.

Total Dietary Habits Score	Coef.	Std. Err.	*t*	*p* > |*t*|	(95% Conf. Interval)	
Food attitudes score	1.07	0.07	15.22	0.000 *	0.93	1.21
Food security score	1.06	0.15	7.22	0.000 *	0.77	1.34
Sex: female	0.97	0.37	−2.62	0.009 *	−1.69	−0.24
Ethnicity	−0.74	0.19	−3.98	0.000 *	−1.10	−0.37
Residence	−0.06	0.06	−0.96	0.34	−0.18	0.06
Education	0.07	0.09	0.73	0.46	−0.11	0.25
Employment	−0.06	0.14	−0.42	0.67	−0.33	0.21
Marital status	0.26	0.14	1.91	0.06	−0.01	0.53
% of time spent at home	1.26	0.28	4.45	0.000 *	0.70	1.81
Age range	−1.02	0.13	−8.08	0.000 *	−1.26	−0.77
Household size	−0.07	0.12	−0.57	0.57	−0.31	0.17
BMI	0.06	0.02	2.73	0.006 *	0.02	0.11
Weight change	0.60	0.25	2.39	0.017 *	0.11	1.10
Medical conditions	−0.01	0.04	−0.35	0.73	−0.09	0.06
Tried a diet	0.88	0.48	1.86	0.06	−0.05	1.82
Nutritional supplement intake	2.16	0.43	5.05	0.000 *	1.32	3.00
Total activity score	1.14	0.07	16.27	0.000 *	1.00	1.28
Food attitudes * Food security	−0.10	0.03	−3.58	0.000 *	−0.16	−0.05

Note. * *p* < 0.05.; Coef = coefficient; Std. Err. = standard error; *t =* coefficient divided by its standard error; Conf. = confidence; BMI = Body Mass Index.

## Supplementary Materials

The following are available online at https://www.mdpi.com/2072-6643/12/11/3468/s1, File S1: Survey on U.S. Adults’ Dietary habits, Food Attitudes and Food security status during COVID-19.
