# Supplementary material for: Relationship between Dietary Habits, Food Attitudes and Food Security Status among Adults Living within the United States Three Months Post-Mandated Quarantine: A Cross-Sectional Study
Source: Nutrients. 2020 Nov 12;12(11):3468. doi: 10.3390/nu12113468 (PMC7697798; doi:10.3390/nu12113468)
Supplement: Supplementary file 1 [file nutrients-12-03468-s001.pdf]

File S1: Survey on U.S. Adults' Dietary habits, Food Attitudes and Food security status during COVID-19

1. **Dietary Habits.** For the following foods listed, indicate if the amount you have consumed has increased, decreased or remained the same since COVID-19

Food Category/ Item	Increased	Decreased	No change
Milk and non-milk alternatives, yogurt and cheese			
Margarine or butter			
Fruit (fresh, frozen or canned)			
Fruit juice			
Non-starchy vegetables such as broccoli, carrots, green beans (fresh, frozen or canned), salad			
Vegetable or tomato juice			
Eggs, chicken, turkey			
Beef, pork or lamb			
Processed meats such as bacon, hot dogs, sausage, salami, bologna or luncheon meats			
Fish and shellfish			
Cold breakfast cereal			
White bread including pita bread			
Dark bread including pita bread			
French fried potatoes			
Starchy vegetables such as corn, peas, and beans			
White rice or pasta			
Brown rice or whole-grain pasta			
Potato chips or other salty snacks such as crackers			
Nuts or seeds			
Peanut butter or other nut butter spreads			
Sweets – candy, cake, cookies, pies			
Oil such as olive, sunflower			
Water			
Coffee or tea			
Immune enhancing beverages – such as ginger, curcumin			
Beer or wine			
Hard liquor – such as whiskey, rum, vodka, gin			
Low calorie carbonated beverages such as diet colas			
Carbonated beverages with sugar such as colas, lemonade, fruit drinks			

2. **Food Attitudes.** Since COVID-19, indicate if your attitudes towards eating have changed for the following statements:

	Increased	Decreased	No change
I find that when I start eating certain foods, I end up eating much more than planned			
I find myself continuing to consume certain foods even though I am no longer hungry			
I eat to the point where I feel physically ill			
I spend a lot of time feeling sluggish or fatigued from overeating			
I find myself constantly eating certain foods throughout the day			
My behavior with respect to food and eating causes significant distress.			

Health Characteristics and Anthropometrics.

3. What is your current height in feet and inches? For example, 5 feet 8 inches

4. What is your current weight in pounds?

5. Since COVID-19, has your weight:
   a. Increased
   b. Decreased
   c. No change

6. Which of the following conditions do you have? (select all that apply)
   a. Cancer
   b. Dementia / Alzheimer's
   c. Depression
   d. Diabetes (high blood sugar)
   e. Diverticulosis/Diverticulitis
   f. Gastric reflux
   g. Heart disease (includes high blood pressure, heart attack, artery disease, stroke, angina)
   h. Irritable Bowel
   i. Liver disease (cirrhosis, fatty liver)
   j. Lung disease
   k. Nausea/Vomiting
   l. Other (please indicate) ______________
   m. None of the above

7. Since COVID-19, have you tried a diet?
   a. Yes
   b. No

8. If yes, which of the following diets have you tried/currently on:
    a. Low carbohydrate diet
    b. Low fat diet
    c. Low salt diet
    d. Plant-based diet
    e. Weight management diet
    f. Other:

9. Since COVID-19, have you begun taking nutritional supplements?
    a. Yes
    b. No

10. If yes, which of the following nutritional supplements are you currently taking?
    a. Calcium
    b. Magnesium
    c. Multi-vitamin
    d. Iron
    e. Omega 3
    f. Omega 6
    g. Protein (bars, shakes, powder)
    h. Vitamin B complex
    i. Vitamin C
    j. Vitamin D
    k. Other:

11. **Lifestyle Habits.** For the following 8 statements, indicate which activities have increased, decreased, or no change since COVID-19:

	Increased	Decreased	No change
Eating			
Exercising (Pilates, yoga, Running, walking or other cardio activity)			
Physical activity (chores, gardening)			
Reading/Studying			
Sleep hours and quality			
Smoking (Cigarettes, Cigar, Hookah)			
Socialization			
Use of electronic devices			

Food Security. For the following 6 statements, click the best answer since COVID-19:

12. The food that (I/we) bought just didn't last, and (I/we) didn't have money to get more
    a. Often true
    b. Sometimes true
    c. Never true
    d. Don't know or Refused to answer

13. (I/we) couldn't afford to eat balanced meals.
    a. Often true
    b. Sometimes true
    c. Never true
    d. Don't know or Refused to answer

14. Did (you/or other adults in your household) ever cut the size of your meals or skip meals because there wasn't enough money for food?
    a. Yes
    b. No
    c. Don't know

15. How often since COVID-19, has this happen?
    a. The past 2 months
    b. The past month
    c. Only once
    d. Don't know

16. Since COVID-19, did you ever eat less than you felt you should because there wasn't enough money for food?
    a. Yes
    b. No
    c. Don't Know

17. Since COVID-19, were you every hungry but didn't eat because there wasn't enough money for food?
    a. Yes
    b. No
    c. Don't Know

Demographics. Please respond to the following demographic questions.

18. Since COVID-19, you are currently staying at home:
    a. 25% or less
    b. 50-75%
    c. 75% -95%
    d. Never have left my house

19. What is your age range?
    a. 18-24 years old
    b. 25–29 years old
    c. 30-49 years old
    d. 50-59 years old
    e. 60-69 years old
    f. 70 years old or older

20. What is your sex?
    a. Male
    b. Female
    c. Other

21. What is your race/ethnicity?
    a. African American
    b. Asian
    c. Caucasian
    d. Hispanic
    e. Native American
    f. Other: ______________

22. What region of the United States do you reside for at least 8 months of the year?
    a. New England (Connecticut, Maine, Massachusetts, Rhode Island, Vermont)
    b. Mid-Atlantic (New Jersey, New York, Pennsylvania)
    c. South Atlantic (Delaware, Florida, Georgia, Maryland, North Carolina, South Carolina, Virginia, Washington DC, West Virginia)
    d. East North Central (Illinois, Indiana, Michigan, Ohio, Wisconsin)
    e. East South Central (Alabama, Kentucky, Mississippi, Tennessee)
    f. West North Central (Iowa, Kansas, Minnesota, Missouri, Nebraska, North Dakota, South Dakota)
    g. West South Central (Arkansas, Louisiana, Texas)
    h. Mountain (Arizona, Colorado, Idaho, Montana, Nevada, New Mexico, Utah, Wyoming)
    i. Pacific (Alaska, California, Hawaii, Oregon, Washington)

23. Besides yourself, how many people live in the household?

24. What is your education level?
    a. No schooling completed
    b. Nursery school to 8th grade
    c. Some high school, no diploma
    d. High school graduate, diploma or the equivalent (for example: GED)
    e. Some college credit, no degree
    f. Trade/technical/vocational training
    g. Associate degree
    h. Bachelor's degree
    i. Master's degree
    j. Professional degree
    k. Doctorate degree

25. What is your current employment status?
    a. Full-time
    b. Part-time
    c. Unemployed
    d. Other

26. What is your marital status?
    a. Married
    b. Single
    c. Widowed
    d. Divorced
    e. Other
